# Non-contiguous finished genome sequence and description of *Herbaspirillum massiliense* sp. nov.

**DOI:** 10.4056/sigs.3086474

**Published:** 2012-12-15

**Authors:** Jean-Christophe Lagier, Gregory Gimenez, Catherine Robert, Didier Raoult, Pierre-Edouard Fournier

**Affiliations:** 1 Unité de Recherche sur les Maladies Infectieuses et Tropicales Emergentes, Aix-Marseille Université

**Keywords:** *Herbaspirillum massiliense*, genome

## Abstract

*Herbaspirillum massiliense* strain JC206^T^ sp. nov. is the type strain of *H. massiliense* sp. nov., a new species within the genus *Herbaspirillum*. This strain, whose genome is described here, was isolated from the fecal flora of a healthy Senegalese patient. *H. massiliense* is an aerobic rod. Here we describe the features of this organism, together with the complete genome sequence and annotation. The 4,186,486 bp long genome (one chromosome but no plasmid) contains 3,847 protein-coding and 54 RNA genes, including 3 rRNA genes.

## Introduction

*Herbaspirillum massiliense* strain JC206^T^ (= CSUR P159 = DSMZ 25712) is the type strain of *H. massiliense* sp. nov. This bacterium was isolated from the stool of a healthy Senegalese patient. It is a Gram-negative, aerobic, flagellated, indole-negative bacillus.

The current approach to classification of prokaryotes, generally referred to as polyphasic taxonomy, relies on a combination of phenotypic and genotypic characteristics [1]. However, as more than 3,000 bacterial genomes have been sequenced [2], we recently proposed that genomic information should be integrated in the description of new bacterial species [3,4].

The genus *Herbaspirillum* (Baldani *et al*. 1986) was created in 1986 [5,6]. To date, this genus, comprised of nitrogen-fixing, Gram-negative bacilli, contains 13 species and two subspecies, including *H. aquaticum* (Dobritsa *et al.* 2010) [7], *H. aurantiacum* (Carro *et al.* 2011) [8], *H. autotrophicum* (Aragno and Schlegel 1978) Ding and Yokota 2004 [9], *H. canariense* (Carro *et al.* 2011) [8], *H. chlorophenolicum* (Im *et al.* 2004) [10], *H. frisingense* (Kirchhof *et al.* 2001) [11], *H. hiltneri* (Rothballer *et al.* 2006) [12], *H. huttiense* subsp. *huttiense* (Leifson 1962) Ding and Yokota 2004 [9], *H. huttiense* subsp. *putei* (Ding and Yokota 2004) Dobritsa *et al.* 2010 [7], *H. lusitanum* (Valverde *et al.* 2003) [13], *H. rhizosphaerae* (Jung *et al.* 2007) [14], *H. rubrisubalbicans* (Christopher and Edgerton 1930) Baldani *et al.* 1996 [6], *H. seropedicae* (Baldini *et al.* 1986) [5], and *H. soli* (Carro *et al.* 2011) [8]. Members of the genus *Herbaspirillum* have mainly been isolated from the environment, in particular from soil, and from plants for which they play the role of growth promoters, but have also occasionally been isolated from humans, either as proven pathogens, causing bacteremia in leukemic patients [15,16], as potential pathogens in aortic aneurysms [17], or in respiratory secretions from cystic fibrosis patients [18,19]. To the best of our knowledge, this is the first to report the isolation of a *Herbaspirillum* sp. from the normal fecal flora. Here we present a summary classification and a set of features for *H. massiliense* sp. nov. strain JC206^T^ (= CSUR P159 = DSMZ 25712) together with the description of the complete genomic sequencing and annotation. These characteristics support the circumscription of the species *H. massiliense*.

## Classification and features

A stool sample was collected from a healthy 16-year-old male Senegalese volunteer patient living in Dielmo (a rural village in the Guinean-Sudanian zone in Senegal), who was included in a research protocol. Written assent was obtained from this individual; no written consent was needed from his guardians for this study because he was older than 15 years old (in accordance with the previous project approved by the Ministry of Health of Senegal and the assembled village population and as published elsewhere [20].) Both this study and the assent procedure were approved by the National Ethics Committee of Senegal (CNERS) and the Ethics Committee of the Institut Fédératif de Recherche IFR48, Faculty of Medicine, Marseille, France (agreement numbers 09-022 and 11-017). Several other new bacterial species were isolated from this specimen using various culture conditions [3,4].

**Figure 1:**
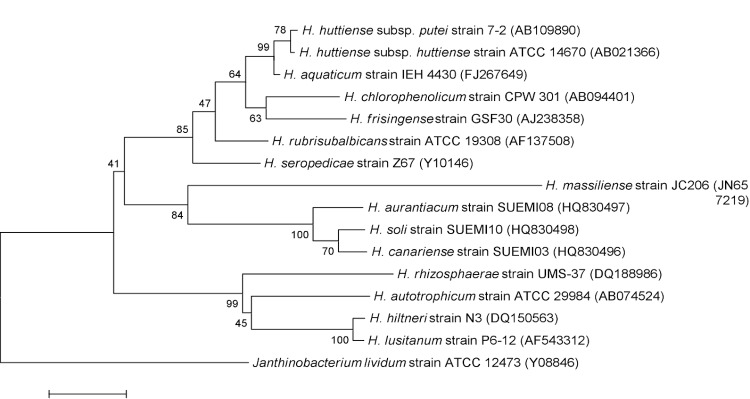
Phylogenetic tree highlighting the position of *Herbaspirillum massiliense* strain JC206^T^ relative to other type strains within the *Herbaspirillum* genus. GenBank accession numbers are indicated in parentheses. Sequences were aligned using CLUSTALW, and phylogenetic inferences obtained using the maximum-likelihood method within the MEGA software. Numbers at the nodes are bootstrap values obtained by repeating 500 times the analysis to generate a majority consensus tree. *Janthinobacterium lividum* was used as an outgroup. The scale bar represents a 0.5% nucleotide sequence divergence.

The fecal specimen was preserved at -80°C after collection and sent to Marseille. Strain JC206^T^ (Table 1) was isolated in June 2011 after passive filtration of the stool sample to select motile species using companion plate, cell culture inserts with 0.4 μm-pore membranes (Becton Dickinson, Heildeberg, Germany) and Leptospira broth (BioMerieux, Marcy l’Etoile, France). Subsequently, we cultivated strain JC206^T^ on 5% sheep blood agar in an aerobic atmosphere at 37°C. This strain exhibited a 96.7% 16S rDNA nucleotide sequence similarity with *H. aurantiacum* (Carro *et al*. 2012), the phylogenetically closest validly published *Herbaspirillum* species (Figure 1), that was cultivated from volcanic soil in Canary Islands. This value was lower than the 98.7% 16S rRNA gene sequence threshold recommended by Stackebrandt and Ebers to delineate a new species without carrying out DNA-DNA hybridization [29].

**Table 1:** Classification and general features of *Herbaspirillum massiliense* strain JC206^T^ according to the MIGS recommendations [21]

**MIGS ID**	**Property**	**Term**	**Evidence code^a^**
	Current classification	Domain: *Bacteria*	TAS [22]
		Phylum *Proteobacteria*	TAS [23]
		Class *Betaproteobacteria*	TAS [24,25]
		Order *Burkholderiales*	TAS [24,26]
		Family *Oxalobacteriaceae*	TAS [24,27]
		Genus *Herbaspirillum*	TAS [5,6]
		Species *Herbaspirillum massiliense*	IDA
		Type strain JC206^T^	IDA
	Gram stain	Negative	IDA
	Cell shape	Rod	IDA
	Motility	Motile	IDA
	Sporulation	Nonsporulating	IDA
	Temperature range	Mesophile	IDA
	Optimum temperature	37°C	IDA
MIGS-6.3	Salinity	Growth in BHI medium + 5% NaCl	IDA
MIGS-22	Oxygen requirement	Aerobic	IDA
	Carbon source	Unknown	
	Energy source	Unknown	
MIGS-6	Habitat	Human gut	IDA
MIGS-15	Biotic relationship	Free living	IDA
MIGS-14	Pathogenicity Biosafety level Isolation	Unknown 2 Human feces	
MIGS-4	Geographic location	Senegal	IDA
MIGS-5	Sample collection time	September 2010	IDA
MIGS-4.1	Latitude	13.7167	IDA
MIGS-4.1	Longitude	– 16.4167	IDA
MIGS-4.3	Depth	Surface	IDA
MIGS-4.4	Altitude	51 m above sea level	IDA

Evidence codes - IDA: Inferred from Direct Assay; TAS: Traceable Author Statement (i.e., a direct report exists in the literature); NAS: Non-traceable Author Statement (i.e., not directly observed for the living, isolated sample, but based on a generally accepted property for the species, or anecdotal evidence). These evidence codes are from the Gene Ontology project [28]. If the evidence is IDA, then the property was directly observed for a live isolate by one of the authors or an expert mentioned in the acknowledgements.

**Figure 2:**
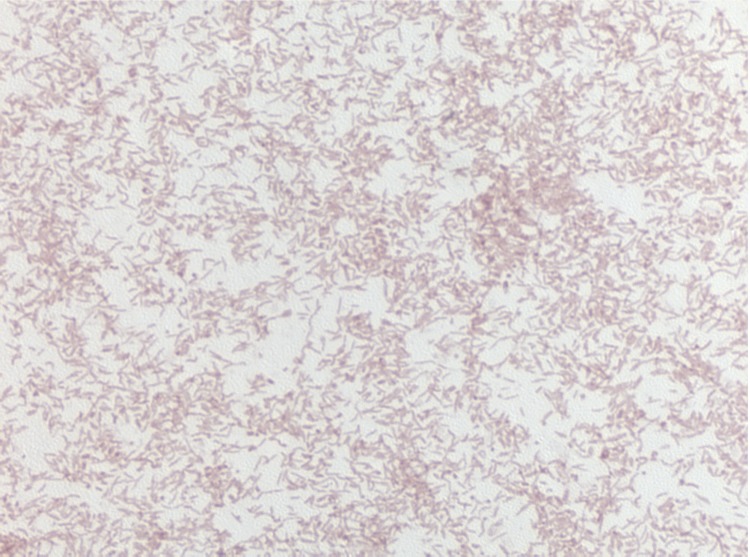
Gram staining of *H. massiliense* strain JC206^T^.

**Figure 3:**
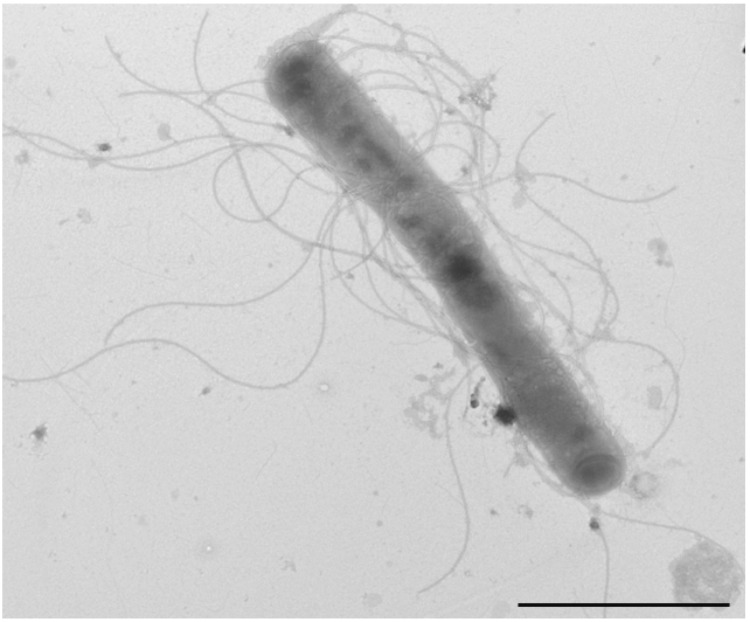
Transmission electron microscopy of *H. massiliense* strain JC206^T^, using a Morgani 268D (Philips) at an operating voltage of 60kV.The scale bar represents 900 nm.

Different growth temperatures (25, 30, 37, 45°C) were tested. No growth occurred at either 25°C or 45°C, growth occurred at either 30 or 37°C. Optimal growth was observed at 37°C. Colonies were light brown, opaque and 0.5 mm in diameter on blood-enriched Columbia agar and Brain Heart Infusion (BHI) agar. Growth of the strain was tested under anaerobic and microaerophilic conditions using GENbag anaer and GENbag microaer systems, respectively (BioMérieux), and in the presence of air, of 5% CO2 and in anaerobic conditions. Optimal growth was obtained aerobically, with weak growth being observed under microaerophilic condition and with 5% CO_2_. No growth occurred under anaerobic conditions. Gram staining showed Gram negative curved rods (Figure 2). A motility test was positive. Cells grown on agar have a mean diameter of 0.44 µm by electron microscopy and have several polar flagella (Figure 3).

Strain 206^T^ exhibited catalase and oxidase activities. Using an API 20 NE strip (BioMerieux), nitrate reduction, indole formation, glucose fermentation and urease were negative. Arginine dihydrolase and esculin hydrolysis were positive. *H. massiliense* is susceptible to ticarcillin, imipenem, trimethoprim/sulfamethoxazole, gentamicin, amikacin, and colimycin but resistant to fosfomycin and nitrofurantoin.

**Figure 4:**
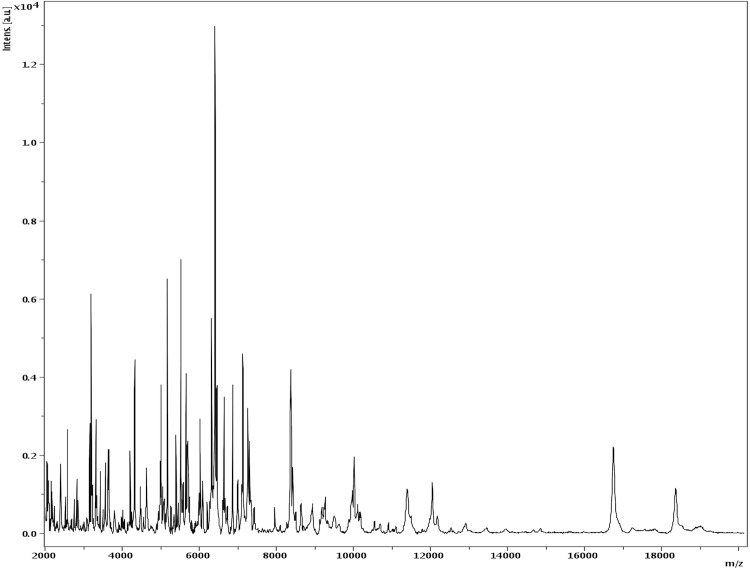
Reference mass spectrum from *H. massiliense* strain JC206^T^. Spectra from 12 individual colonies were compared and a reference spectrum was generated.

Matrix-assisted laser-desorption/ionization time-of-flight (MALDI-TOF) MS protein analysis was carried out as previously described [30] using a Microflex spectrometer (Bruker Daltonics, Germany). Spectra were compared with the Bruker database that contained no spectrum from *Herbaspirillum* species. No significant score was obtained with any other taxon. We incremented our database with the spectrum from strain JC206 ^T^ (Figure 4).

## Genome sequencing information

### Genome project history

The organism was selected for sequencing on the basis of its phylogenetic position and 16S rRNA similarity to other members of the genus *Herbaspirillum*, and is part of a “culturomics” study of the human digestive flora aiming at isolating all bacterial species within human feces. It was the second genome of a *Herbaspirillum* species and the first genome of *H. massiliense* sp. nov. A summary of the project information is shown in Table 2. The Genbank accession number of the genome is CAHF00000000 and consists of 27 contigs. Table 2 shows the project information and its association with MIGS version 2.0 compliance.

**Table 2:** Project information

**MIGS ID**	**Property**	**Term**
MIGS-31	Finishing quality	High-quality draft
MIGS-28	Libraries used	Shot Gun, Paired-end 3 Kb library
MIGS-29	Sequencing platforms	454 GS FLX Titanium
MIGS-31.2	Fold coverage	29×
MIGS-30	Assemblers	Newbler version 2.5.3
MIGS-32	Gene calling method	Prodigal
	Genbank ID	CAHF00000000
	Genbank Date of Release	June 1, 2012
	Project relevance	Study of the human gut microbiome

### Growth conditions and DNA isolation

*H. massiliense* sp. nov. strain JC206^T^ (= CSUR P159, = DSM 25712), was grown aerobically on 5% sheep blood-enriched Columbia agar at 37°C. Cell growth from eight petri dishes (“spread plates”) was resuspended in 4×100µl of G2 buffer (EZ1 DNA Tissue kit, Qiagen). A first mechanical lysis was performed by glass powder on the Fastprep-24 device (MP Biomedicals, USA) during 2×20 seconds. DNA was then incubated with lysozyme for 30 minutes at 37°C and extracted using the EZ 1 Advanced XL BioRobot (Qiagen). DNA was concentrated and purified using the QiAmp kit (Qiagen). The yield and concentration were measured using the Quant-it Picogreen kit (Invitrogen) and the Genios_Tecan fluorometer at 52.5 ng/µl.

### Genome sequencing and assembly

Both a shotgun and a 3-kb paired end sequencing were performed on a 454 GS FLX pyrosequencer. Both projects were loaded on a ¼ and a 1/8 regions of a PTP Picotiterplate. The shotgun library was constructed with 500 ng DNA as recommended by the manufacturer (Roche). For paired end sequencing, five µg of DNA were mechanically fragmented using the Hydroshear device (Digilab, Holliston, MA) with an enrichment size at 3-4kb. The DNA fragmentation was visualized using the BioAnalyzer 2100 on a DNA labchip 7500 (Agilent) with an optimal size of 3.944 kb. The library was constructed according to the 454 GS FLX Titanium paired end protocol. Circularization and nebulization were performed and generated a pattern with an optimal at 418 bp. After PCR amplification through 15 cycles followed by double size selection, the single stranded paired end library was then quantified on the Quant-it Ribogreen kit (Invitrogen) on the Genios Tecan fluorometer at 128 pg/µL. The library concentration equivalence was calculated as 5.62 × 10^8^ molecules/µL. The library was stored at -20°C until further use.

The library was clonally amplified with 2 cpb and 3 cpb, respectively, in 2 × 8 emPCR reactions with the GS Titanium SV emPCR Kit (Lib-L) v2 (Roche). The yields of the emPCR were 13.75 and 2.65% for the shotgun and paired end strategies, respectively.

Approximately 790,000 beads were loaded on the GS Titanium PicoTiterPlate PTP Kit 70×75 and sequenced with the GS FLX Titanium Sequencing Kit XLR70 (Roche). The run was performed overnight and then analyzed on the cluster through the gsRunBrowser and Newbler assembler (Roche). A total of 504,311 passed filter wells were obtained and generated 4.69 Mb with a length average of 312 bp. The passed filter sequences were assembled Using Newbler with 90% identity and 40 bp as overlap. The final assembly identified 5 scaffolds and 27 contigs (>100 bp).

### Genome annotation

Open Reading Frames (ORFs) were predicted using Prodigal [31] with default parameters but the predicted ORFs were excluded if they were spanning a sequencing GAP region. The predicted bacterial protein sequences were searched against the National Center for Biotechnology Information (NCBI) nonredundant (NR) and the Clusters of Orthologous Groups (COG) databases using BLASTP. The tRNAScanSE tool [32] was used to find tRNA genes, whereas ribosomal RNAs were found by using RNAmmer [33] and BLASTn against the NR database. ORFans were identified if their BLASTP *E*-value were lower than 1e-03 for alignment length greater than 80 amino acids. If alignment lengths were smaller than 80 amino acids, we used an *E*-value of 1e-05. Such parameter thresholds have already been used in previous works to define ORFans.

## Genome properties

**Figure 5:**
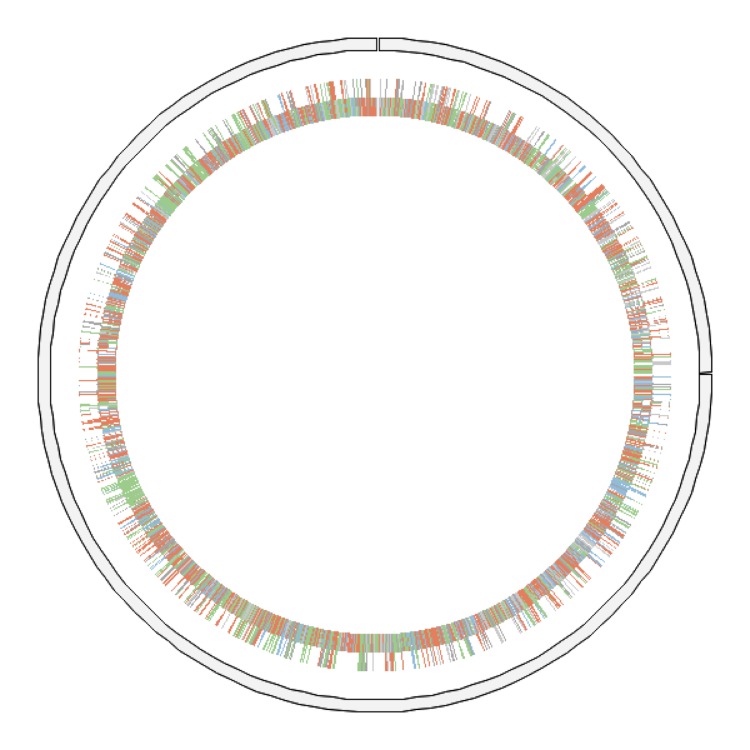
Graphical circular map of the chromosome. Genes are colored according to their COG categories as follows: information storage and processing (blue), cellular processing and signaling (green), metabolism (red) and poorly characterized (grey).

The genome is 4,186,486 bp long (one chromosome but no plasmid) with a 59.73% GC content (Table 3 and Figure 5). Of the 3,901 predicted genes, 3,847 were protein-coding genes, and 54 were RNAs. A total of 2,924 genes (74.95%) were assigned a putative function. ORFans accounted for 312 (8.0%) of the genes. The remaining genes were annotated as hypothetical proteins. The distribution of genes into COGs functional categories is presented in Table 4. The properties and the statistics of the genome are summarized in Tables 3 and 4.

**Table 3:** Nucleotide content and gene count levels of the genome

**Attribute**	**Value**	% of total^a^
Genome size (bp)	4,186,486	
DNA coding region (bp)	3,655,584	87.32
DNA G+C content (bp)	2,500,588	59.73
Total genes	3,901	100
RNA genes	54	1.4
Protein-coding genes	3,847	98,61
Genes with function prediction	2,924	74.95
Genes assigned to COGs	3,135	80.36
Genes with peptide signals	378	9.68
Genes with transmembrane helices	955	24.48

^a^The total is based on either the size of the genome in base pairs or the total number of protein coding genes in the annotated genome.

**Table 4:** Number of genes associated with the 25 general COG functional categories

**Code**	**Value**	**%age**^a^	**Description**
J	183	4.69	Translation
A	2	0.05	RNA processing and modification
K	199	5.10	Transcription
L	183	4.69	Replication, recombination and repair
B	2	0.05	Chromatin structure and dynamics
D	45	1.15	Cell cycle control, mitosis and meiosis
Y	0	0	Nuclear structure
V	44	1.13	Defense mechanisms
T	234	6.00	Signal transduction mechanisms
M	272	6.97	Cell wall/membrane biogenesis
N	142	3.64	Cell motility
Z	0	0	Cytoskeleton
W	0	0	Extracellular structures
U	141	3.61	Intracellular trafficking and secretion
O	168	4.31	Posttranslational modification, protein turnover, chaperones
C	230	5.90	Energy production and conversion
G	192	4.92	Carbohydrate transport and metabolism
E	263	6.74	Amino acid transport and metabolism
F	67	1.72	Nucleotide transport and metabolism
H	141	3.61	Coenzyme transport and metabolism
I	176	4.51	Lipid transport and metabolism
P	147	3.77	Inorganic ion transport and metabolism
Q	93	2.38	Secondary metabolites biosynthesis, transport and catabolism
R	444	11.38	General function prediction only
S	384	9.84	Function unknown
-	95	2.44	Not in COGs

The total is based on the total number of protein coding genes in the annotated genome.

## Comparison with *Herbaspirillum seropedicae*

To date, the genome from *H. seropedicae* strain SmR1 is the only genome from *Herbaspirillum* species that has been sequenced [34]. By comparison with *H. seropedicae*, *H. massiliense* exhibited a smaller genome (4,186,486 bp *vs* 5,513,887 bp, respectively), a lower G+C content (59.73% *vs* 63.4%, respectively) and a smaller number of genes (3,901 *vs* 4,804). In contrast, *H. massiliense* had higher ratios of genes per Mb (0.93 *vs* 0.87) and genes with assigned functions (74.9% *vs* 64.7%).

## Conclusion

On the basis of phenotypic, phylogenetic and genomic analyses, we formally propose the creation of *Herbaspirillum massiliense* sp. nov. that contains the strain JC206^T^. This bacterium has been found in Senegal.

### Description of *Herbaspirillum massiliense* sp. nov.

*Herbaspirillum massiliense* (mas.il.ien’se. L. gen. neutr. n. *massiliense*, of Massilia, the Latin name of Marseille where strain JC206^T^ was cultivated).

Colonies are 0.5 mm in diameter on blood-enriched Columbia agar and Brain Heart Infusion (BHI) agar. Cells are rod-shaped with a mean diameter of 0.44 µm. Motile with tufts of polar flagellae optimal growth occurs under aerobic conditions. Weak growth is observed under microaerophilic conditions and with 5% CO_2_. No growth is observed under anaerobic conditions. Growth occurs between 30-37°C, with optimal growth observed at 37°C.

Cells stain Gram-negative. Catalase, oxidase and arginine dihydrolase activities, as well as esculin hydrolysis are present. Nitrate reduction and indole production are absent. Cells are susceptible to ticarcillin, imipenem, trimethoprim/sulfamethoxazole, gentamicin, amikacin, and colimycin. The G+C content of the genome is 59.73%. The 16S rRNA and genome sequences are deposited in Genbank under accession numbers JN657219 and CAHF00000000, respectively. The type strain JC206^T^ (= CSUR P159 = DSMZ 25712) was isolated from the fecal flora of a healthy patient in Senegal.
